# Minnesota State Medical Society

**Published:** 1872-02-15

**Authors:** 


### MINNESOTA STATE MEDICAL SOCIETY.

The fourth annual meeting of the Minnesota State Medical Society was held in St. Paul commencing Monday, Feb. 6th, 1872. About sixty members were in attendance, and the reports and papers read were of an unusually interesting and important character.

Dr. C. Hill, from the Committee on Medical Jurisprudence, made a report in which he maintained that when a medical man was called to appear as a witness to give his opinion as an expert, he should be paid a suitable fee for the same. The paltry sum paid to witnesses generally was undignified, and the whole medical fraternity ought to set its face against testifying for such a sum.

Dr. C. N. Hewett presented an interesting report from the Committee on Surgery.

Dr. A. J. Stone, for the Committee on Medical Education, presented a highly important report, in which the following recommendations were embodied:

First. The appointment by each State Society of a Board of Examiners, who are to examine such person who desires to study medicine; and that the standard of qualification shall be as high as that of any literary institution.

Second. That no person shall be received as a student in any medical college or by any physician, till he can furnish a satisfactory certificate from some literary or scientific institution of known good standing.

Third. That each college adopt a progressive course of study, of four years duration, to be divided into four yearly courses, each year to be divided into two terms of not less than sixteen weeks each, with an examination at the close of each year.

Fourth, makes provisions for entering an advanced class. The whole senior must be spent at the college.

Fifth, points out what studies should be pursued.

Sixth, makes provisions for a faculty of a dozen full chairs.

Seventh, provides for an adjunct faculty to teach enumerated studies.

Eighth, declares that the medical department should be located in the largest medical center of the State, without reference to the other university buildings.

Ninth, provides for the granting of the degrees of M.B. and M.D.

The report declares that as to fees it is as much the duty of the State to furnish a free professional education to the children as it is to offer a free classical, scientific, or agricultural one. If there are to be fees they should be fixed at the lowest possible amount.

The twelfth recommendation is the most significant. It is that the State Normal Board be empowered to employ some regular physician to give instructions, so far as is practicable, during each session of each Normal School, in the rudiments of Anatomy and Physiology, and that all Superintendents of schools employ some regular physician to perform the same duty in each of the district and graded schools in the State.

The following reports were also read during the first day's session: Report of the Committee on Climatology and Epidemics, read by Dr. Sweeney; A Report upon Ulcers, by H. H. Kimball, M.D., of Minneapolis; an interesting report on Obstetrics and Gynæcology, by W. L. Lincoln, M.D.; and a report on Materia Medica, by Dr. W. F. Hutchinson.

The retiring President, Dr. Franklin Staples, delivered an eloquent address on "The Duties and Obligations of the Medical Profession."

The election of officers for the ensuing year resulted as follows: President — W. W. Mayo, M.D., of Rochester; Vice Presidents — W. W. Sweeney, M.D., Red Wing, S. C. McCormick, M.D., Duluth, W. L. Lincoln, M. D., Wabashaw; Treasurer—S. B. Sheardown, M.D., Stockton; Secretary — Chas. E. Smith, M. D., St. Paul.

At the evening session the following resolution was introduced by Dr. Adams:

*Resolved*, That the Legislative Assembly of the State of Minnesota is hereby respectfully requested to pass a law legalizing dissection by the medical profession of the State of Minnesota, and that the Secretary of this Society be directed to deliver a copy of this resolution, with the seal of the said Society, and the signatures of the proper officers duly affixed thereto, to the Judiciary Committee of the Assembly.

Dr. Stone reported that the Committee on Anatomy had arranged with Representative Sabin to introduce a bill to legalize dissection. The matter would be brought before the Judiciary Committee at its next meeting, and there was little doubt but that satisfactory arrangements would be entered into.

At the opening of the second day's session Dr. Mayo, the newly-elected President, delivered a highly interesting and appropriate inaugural address. The Annual Essay was then read by Dr. Levi P. Dodge, and also an interesting report upon Small Pox by Dr. Hagan.

In the evening the members of the Society and invited guests assembled at the Merchant's Hotel to partake, at the invitation of the Ramsey County Medical Society, of a sumptuous banquet.

The highly prosperous and satisfactory condition of the Soceity is shown by the following statements taken from the address of the retiring President, Dr. Staples:

As members of the Minnesota State Medical Society we may congratulate ourselves upon the prosperous condition of our Society. Although our present organization has had an existence of only three years, its membership now numbers one hundred and forty-three, twenty-three new members having been added at the last annual meeting, and fifteen at the last semi-annual meeting.

We have to record the death of two members during the year, that of Dr. E. Herman Smith, the former efficient Secretary of this Society, and Dr. Perry Chamer. Dr. Smith died at his family home in Connecticut, on the 12th of February last, of phthisis pulmonalis. He was greatly beloved by his professional brethren, and a worthy member of this Society. Dr. Chamer, although a worthy and esteemed member, was less known to the profession of the State, having been elected a member of our Society at the meeting in June.

Owing to the character of the men who make up the regular medical profession of Minnesota, our State Society has to contend less with the obstacles in the way of progress than the societies of some of the older States.

Looking from the standpoint from which I have been permitted to observe this Society during the past year, I have beheld only a united, harmonious, and active membership of intelligent and educated physicians.

The number of standing committees as created by the constitution are, of course, the same as in former years.

Four new special committees have been added to the number of last year, making the whole number, at present, nine, whose reports you have heard.

Omaha Medical Society. — At the annual meeting of the Omaha Medical Society, held on the evening of Feb. 13th, 1872, the following officers were elected for the ensuing year: President — H. P. Mathewson, M.D.; Vice Pres. — R. C. Moore, M.D.; Recording Sec'y — Geo. Tilden, M. D.; Corresponding Sec'y — M. T. Anderson, M.D.; Treasurer — H. R. Benjamin, M.D.

